# Parasitic outbreak of the copepod *Balaenophilus manatorum* in neonate loggerhead sea turtles (*Caretta caretta*) from a head-starting program

**DOI:** 10.1186/s12917-017-1074-8

**Published:** 2017-06-02

**Authors:** J. L. Crespo-Picazo, D. García-Parraga, F. Domènech, J. Tomás, F. J. Aznar, J. Ortega, J. M. Corpa

**Affiliations:** 1 Veterinary Services, Fundación Oceanogràfic de la Comunitat Valenciana, Eduardo Primo Yúfera 1B, 46013 Valencia, Spain; 2 Marine Zoology Unit, Cavanilles Institute of Biodiversity and Evolutionary Biology, University of Valencia, Catedrático José Beltrán 2, 46980 Paterna, Valencia Spain; 3 Biomedical Sciences Research Institute, PASACTA (Histología y Anatomía Patológica), Faculty of Veterinary Medicine, Universidad CEU Cardenal Herrera, Avda. Seminario, s/n, 46113 Moncada, Valencia Spain

**Keywords:** *Caretta caretta*, Sea turtle, Head-starting, *Balaenophilus manatorum*, Parasitic infestation, Ectoparasite, Outbreak

## Abstract

**Background:**

Diseases associated to external parasitosis are scarcely reported in sea turtles. During the last decades several organism have been documented as a part of normal epibiont community connected to sea turtles. The copepod *Balaenophilus manatorum* has been cited as a part of epibiont fauna with some concern about its parasitic capacity. This study serves three purposes, i.e. (i) it sheds light on the type of life style that *B. manatorum* has developed with its hosts, particularly turtles; (ii) it makes a cautionary note of the potential health risks associated with *B. manatorum* in sea turtles under captivity conditions and in the wild, and (iii) it provides data on effective treatments against *B. manatorum*.

**Results:**

We report for the first time a massive infestation of the copepod *B. manatorum* and subsequent acute mortality in a group of loggerhead sea turtle hatchlings. Four-month-old turtles from a head-starting program started exhibiting excitatory and fin rubbing behavior preceding an acute onset of lethargy, skin ulceration and death in some animals. All the individuals (*n* = 57) were affected by severe copepod load and presented different degrees of external macroscopic skin lesions. The ventral area of front flippers, axillar and pericloacal skin were mostly affected, and were the main parasite distribution regions. Copepods were also detected on plastron and carapace sutures. The gut contents of *B. manatorum* reacted positively for cytokeratin, indicating consumption of turtle skin. Severe ulcerative necrotic dermatitis and large amount of bacteria presence were the major histopathological findings.

**Conclusions:**

Individual fresh water immersion for 10 min and lufenuron administration (0.1 ppm) to the water system every 2 weeks proved effective for removing turtle parasites and to control re-infestation, respectively. The results from our study clearly indicated that *B. manatorum* individuals consume turtle skin. The pathological effects of this agent and the potential implications in sea turtle conservation and management are discussed.

## Background

Sea turtle populations have dramatically and globally declined in recent decades. Currently 6 of 7 sea turtle species are listed as Vulnerable, Endangered or Critically Endangered according to the International Union for Conservation of Nature (IUCN) Red List [1].

Head-starting programs have been implemented as a complementary tool to recover populations of endangered species, including sea turtles [2]. Head-starting is the practice of growing hatchlings in captivity to maximize survival in the first development stages when mortality rates are higher. Individuals are then released into the sea once they present a pre-established size considered large enough to minimize post-hatchling vulnerabilities [3–5]. Although the use of head-starting programs as a management tool to recover threatened populations has been criticized [6], these programs provide valuable information about species, including growth models and health status after hatching. They are also considered a valuable method for raising public awareness through displays and releases [3].

However, hatchlings reared in captivity may suffer from different problems after or during the head-starting process. Disease outbreaks have been described in sea turtles reared in captivity due to bacterial, viral, parasitic and fungal diseases, as well as traumatic injuries, usually with high morbidity and variable mortality rates [7–10]. Skin lesions could result from the primary pathogenic action of these agents, or the secondary outcome of opportunistic agents after immunosuppressive events or traumatic injuries. In this study we document a mortality outbreak of sea turtles caused by a crustacean ectoparasite.

Species of the genus *Balaenophilus* are harpacticoid copepods that have developed obligate associations with marine tetrapods worldwide [11, 12]. Currently, the genus includes two species. *Balaenophilus unisetus* occurs on the baleen plates of whales in the Atlantic and Pacific Oceans [13]. *B. unisetus* is considered commensal because it feeds on baleen tissue (perhaps to benefit from its associated microscopic epibiota), but does not harm its hosts [14, 15]. The second species, *B. manatorum*, has been reported on the skin of some sea turtle species in the Atlantic and Pacific Oceans, and in the Mediterranean Sea [11, 16–18] and on the skin of manatees, *Trichechus manatus*, in the western Caribbean [19, 20]. Anatomical adaptations such as modified appendages for strong clasping in all development stages and grasping mouthpart of species of *Balaenophilus* suggest an obligate association with their hosts [12]. Despite copepodites and adults have appendages that could be involved in swimming *Balaenophilus* species seem to spend their entire life on their host [16, 21] even though those features could facilitate transmission through short distances [21].

The association of *B. manatorum* with its hosts is more controversial. In sea turtles, Ogawa et al. [16] and Badillo [15] have provided prima facie evidence that this species may feed on skin and produce epidermis erosion, particularly with high parasitic loads. In manatees, however, Suárez-Morales et al. [20] found no gross evidence of skin damage and hypothesized that *B. manatorum* could feed on suspended particles produced when the manatee feeds.

In this paper, we evaluate the association between *B. manatorum* and its host from an acute infestation outbreak that caused skin disease and mortality among the hatchlings of a loggerhead sea turtle, *Caretta caretta*, from a head-starting program. The pathological effects of this agent and the potential implications in sea turtle conservation and management are discussed.

## Methods

### Animals

All the sea turtles included in this project were authorized by the *Conselleria d’Agricultura, Medi Ambient, Canvi Climàtic i Desenvolupament Rural* of the Regional Valencia Government in a collaborative official agreement with the Oceanogràfic Aquarium of the *Ciudad de las Artes y las Ciencias of Valencia* (east Spain) for rehabilitation and posterior release of animals, and for the postmortem examination of dead individuals.

In June 2014, a loggerhead nest with 131 eggs (two of them were already broken) was found in Alicante (east Spain) in the Western Mediterranean basin, an area beyond the regular nesting range for the species in the Mediterranean Sea [22]. Given the sporadic characteristic of this nesting event, and the fact that the nest was found on a highly developed beach, the clutch was carefully removed and relocated for its protection. The clutch was divided into two egg subsets; one part (89 eggs) was relocated to a natural protected beach in the province of Valencia, and the other part (40 eggs) was kept in artificial incubation at the marine rehabilitation center of the Oceanogràfic aquarium. 102 hatchlings from both places were initially reared at the aquarium facilities under a head-starting program. 30 turtles died due to intestinal bacteria outbreak during second month of life and 15 were moved to other rescue center. The parasitic outbreak occurred 4 months after hatching. At the time of the outbreak, mean straight carapace length ± SD (95% CI) [range] was 5.91 ± 0.51 [4.6–6.84] cm and mean body weight was 43.13 ± 8.7 [21.6–57.4] g.

### Facilities

Sand-filtered seawater was piped through two filtration systems prior to the holding tanks. Sand filters, protein skimmers, ultraviolet light, biological filtration and ozone were used to maintain water quality. Water temperature was typically set at 25 °C (± 1) and a full light spectrum was provided by hydrargyrum quartz iodide (HQI) lightning. Neonates were kept separated from one another in plastic-mesh floating cages to prevent bite wounds and to allow close monitoring of individuals. In addition to the head started turtles, larger injured sea turtles undergoing rehabilitation were also housed at the aquarium. The turtles from the head-starting program and the animals in rehabilitation were kept in separate tanks, but shared the filtration system. Occasionally, and due to space constraints, neonates were maintained in their floating cages inside a tank where an animal being rehabilitated was also kept.

### Treatment

Prior to the outbreak, no preventive treatment was administered when animals were admitted from the rehabilitation center or from the head-starting program. After detecting massive copepod infestations in neonates, all the individuals were immediately treated in a tap water bath for 10 min to remove the parasite load [23]. Additionally, three different experimental treatments were subsequently tested to remove parasites from the filtration system: (1) during week 1, formalin (Formol 40%, methyl aldehyde solution, Guinama S.L.U., Valencia, Spain) was added as follows: 0.015 ml l-1 (day 1); 0.01 ml l-1 (day 3); 0.001 ml l-1 (day 5). Ozone and ultraviolet filter was switched off during treatment and switched on again on day 7. Ten per cent of water was renewed in the system on days 1, 3 and 5. (2) During weeks 2 and 3, the filtration system was treated with chlorine (sodium hypochlorite, 150 g l-1, New Chem S.L., Alicante, Spain), which reached 0.6 ppm of free chlorine. (3) During week 4, a single dosage of 0.1 ppm of lufenuron (Program® 400, Novartis Sanidad Animal S.L., Barcelona, Spain) was applied to the system and repeated every 14 days; i.e., two additional treatments. Daily water replacement was 10% of the total system volume. To ensure that these treatments were not toxic for turtle hatchlings, three individuals were treated following the doses used in fish [24].

### Samples

Sediment of freshwater baths of turtles was used to collect and identify parasites. Parasite specimens were collected and preserved in ethanol 70% for species identification. Complete necropsy was performed on each deceased individual within 6 h postmortem. Multiple tissues, including skin, fat, skeletal muscle, thymus, thyroid, heart, lung, liver, esophagus, stomach, intestine, spleen, pancreas, kidney, gonad, adrenal gland, salt gland and brain, were collected and fixed in 10% neutral formalin. All the tissues were routinely processed for histological examination and stained with hematoxylin and eosin (H&E) staining. To analyze gut contents of in situ *B. manatorum* immunohistochemical staining was undertaken for cytokeratin detection (monoclonal anti-cytokeratin, Isotype IgG1 Kappa, Clone AE1/AE3, Dako) using the avidin-biotin-peroxidase complex (ABC) method as recommended by the manufacturer. Swabs for bacteriology were taken aseptically from the coelomic cavity and the liver, lung and brain sections were frozen at −80 °C. Turtles behavior was checked there times a day as well as during feeding and treatment times. Animals were weighted once a week during the whole duration of the head-starting program.

## Results

**Fig. 1:**
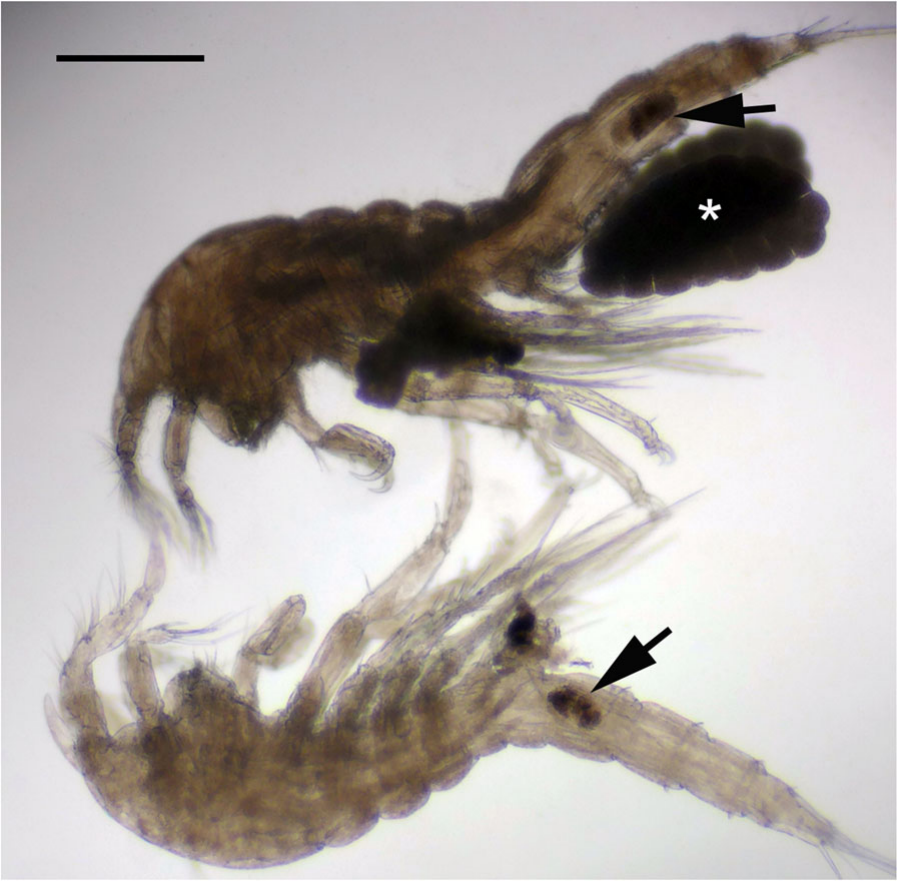
Adult specimens of *B. manatorum* under light microscope examination. Upper individual presents a pair of egg sacs (*asterisk*). Both parasites show *black-brownish* material in the digestive system (*arrows*). Scale bar = 200 μm

**Fig. 2:**
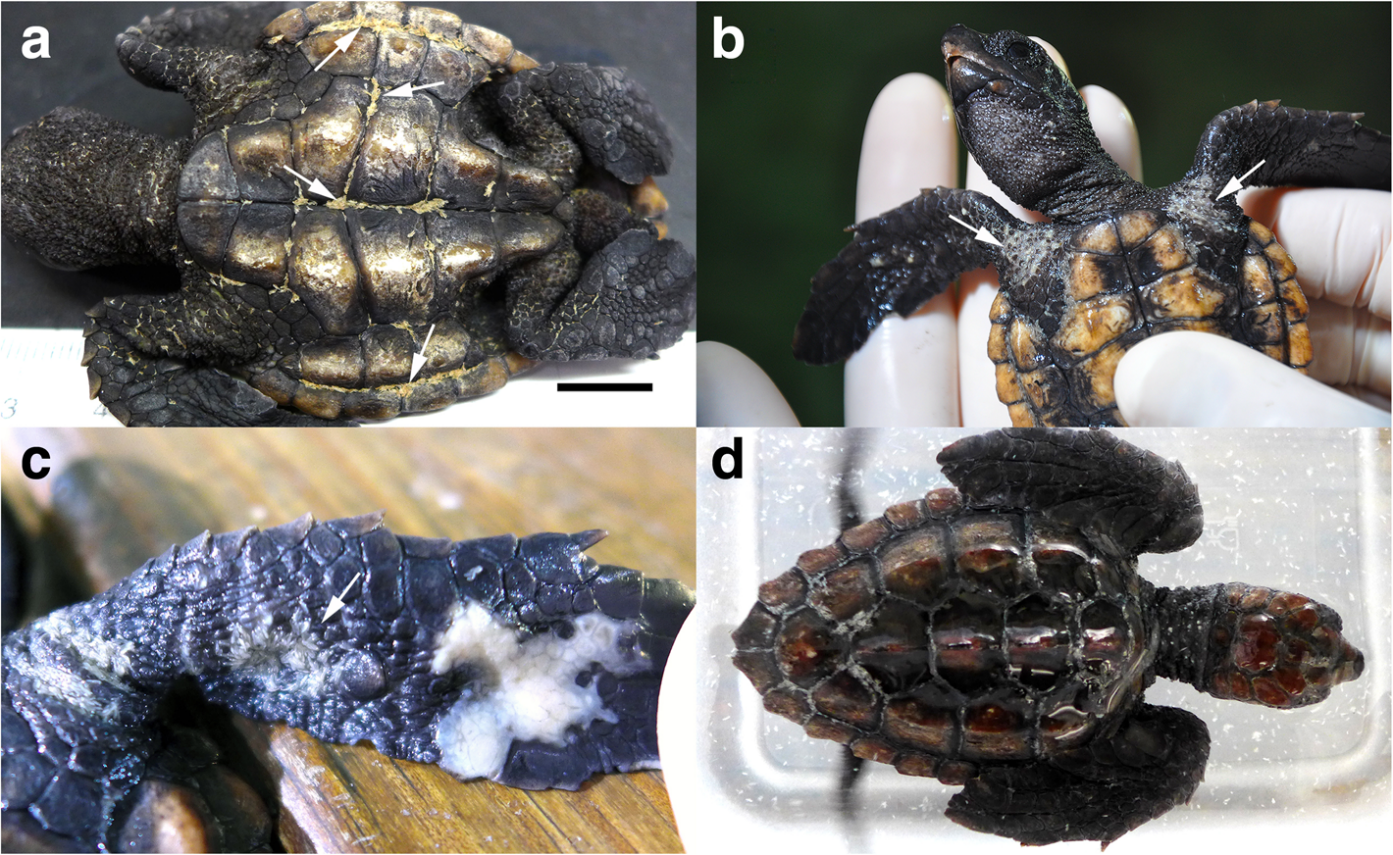
Parasites (*arrows*) were mainly located on the ventral surface of animals: plastron sutures (**a**) pectoral girdle (**b**) and flippers (**c**). Parasites were removed when neonate turtles were immersed in tap water (**d**). Scale bar = 1 cm

Several developmental stages of *Balaenophilus manatorum*, including nauplii, copepodids III to V and male and female adults [16], were collected and identified from all the neonates included in the head-starting program (Fig. 1). All the turtles infested with severe parasite loads (mean intensity (number of parasites per turtles) ± SD (95% CI) [range] was 337.4 ± 212.9 (249.7–462.5) [17–1423], *n* = 43 turtles [21]). Parasites occurred mainly on the ventral surface of flippers, pectoral girdle, pericloacal skin and plastron sutures. In the most severe infestations, copepod accumulations provoked erosion on carapace scutes (Fig. 2).

Clinical signs consisted of acute rubbing behavior (front and rear flippers against the plastron), anorexia, weight lost and lethargy compared with previous weeks. Open wounds and thickened whitish scars with ill-defined margins were present on the ventral surface of flippers, pectoral girdle and pericloacal skin, but smaller lesions were also noticed around the neck, and even on eyelids. In three animals, irregular keratin defects were detected on shell scutes after copepod removal. Six of the neonates (10.5%) died during the 24 h following the onset of clinical signs.

**Fig. 3:**
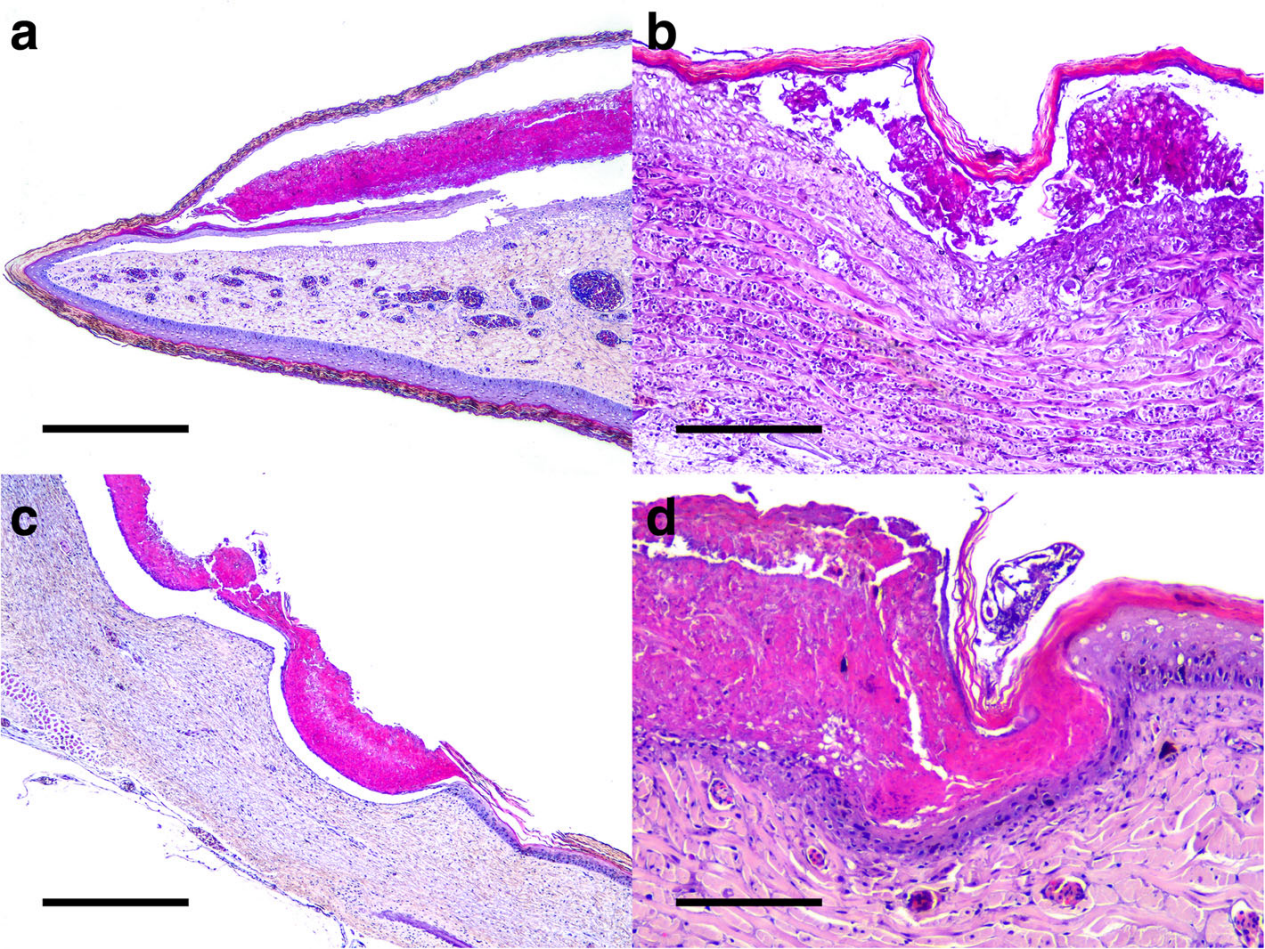
**a** Flipper. Epidermal necrosis with subcorneal blistering. Hematoxylin and eosin; scale bar = 500 μm. **b** Flipper. Epidermal hyperplasia with swelling of keratinocytes and necrosis. Hematoxylin and eosin; scale bar = 200 μm. **c** Carapace. Transepidermal necrosis with characteristic detached eosinophilic amorphous material. Hematoxylin and eosin; scale bar = 500 μm. **d** Flipper. Necrotizing dermatitis with intralesional parasites. Hematoxylin and eosin; scale bar = 200 μm

**Fig. 4:**
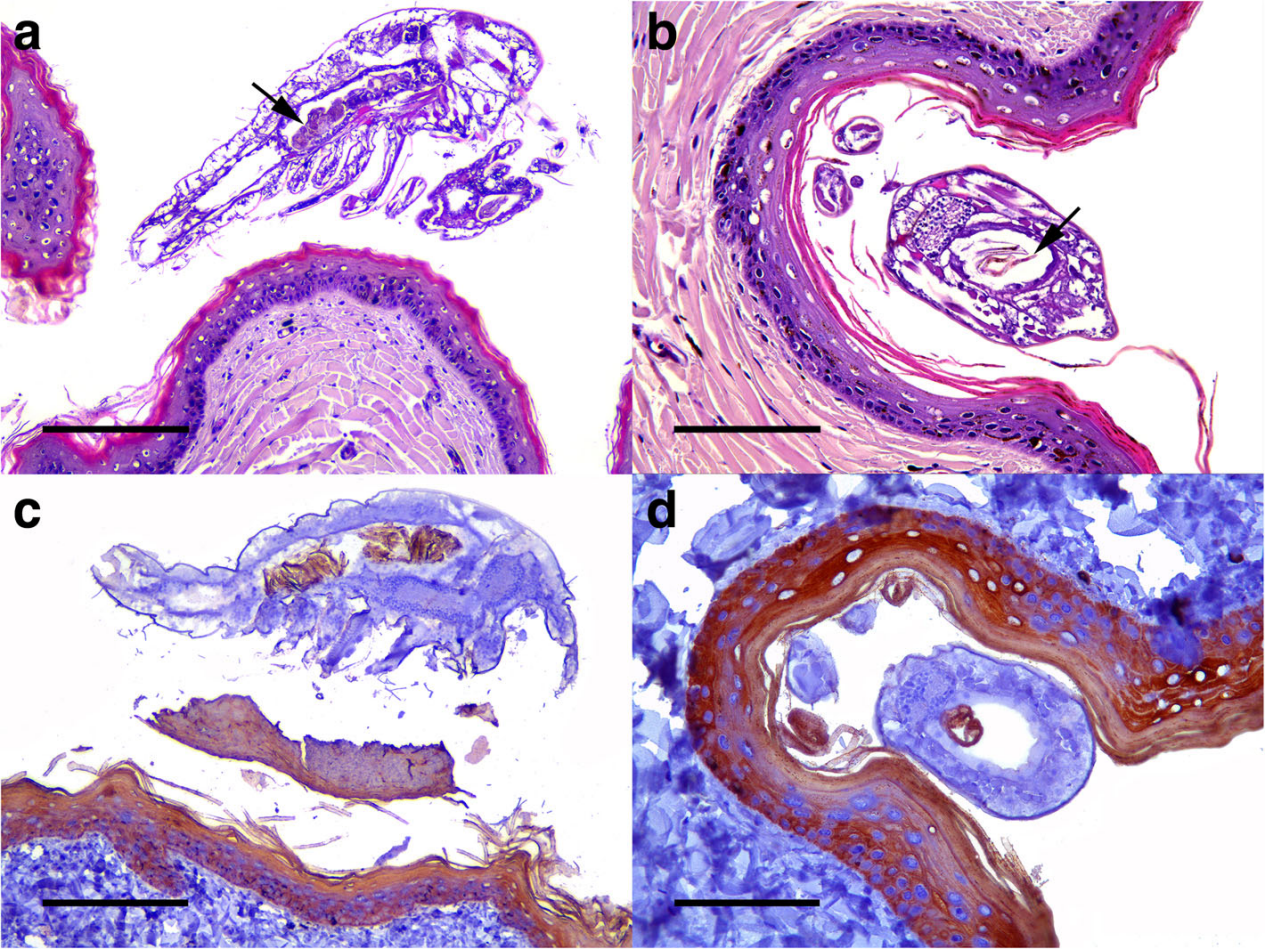
**a** Skin of a flipper. Parasite situated over the epidermis showing abundant granulated *brownish* material into its gut (*arrow*). Hematoxylin and eosin; scale bar = 200 μm. **b** Skin of a flipper. A parasite attached to the keratinized layer of the epidermis. The transversal section of the parasite allows observing *brown* laminated material that resembles keratin and small *black spots* that resemble melanin granules in gut content (*arrow*). Hematoxylin and eosin; scale bar = 100 μm. **c** Longitudinal and **d** cross sections of a parasite. Gut content reacts positively for cytokeratin. Anti-cytokeratin monoclonal antibody and ABC; scale bars = 200 and 100 μm, respectively

Histopathological findings included severe ulcerative necrotizing dermatitis, with associated inflammation and bacteria. Necrosis extended from the epidermis to the superficial dermis, with characteristic detached eosinophilic amorphous material. Multiple intraepidermal pustules consisting of necrotic keratinocytes and heterophils were observed. Other areas presented a separation between the epidermis and dermis with blister formation. Different degrees of inflammation, necrosis and keratin loss were detected on skin. Numerous bacterial colonies were observed on the most superficial necrosis layer in some animals. In several sections, *B. manatorum* individuals were observed as being attached to the epidermis and were associated with a thinner keratin/epidermal layer compared to the adjacent tissue (Fig. 3). These *B. manatorum* showed acidophilic material in the gut that resembled keratin, as well as small black spots that resembled melanin granules, as observed on normal turtle skin. The *B. manatorum* specimens had food pellets composed of light brown to black material in the digestive tract (Fig. 1). Immunohistochemistry indicated that the pellet reacted positively with cytokeratin antibody, which confirmed the presence of keratin in the gut of parasites (Fig. 4). No histopathological lesions were found in sea turtle internal organs. No bacterial growth was obtained from coelomic cavity swabs cultures.

Freshwater baths apparently had an immediate effect on *B. manatorum* by killing a number of individuals, which were observed in the sediment of the treatment tanks (Fig. 2). However, severe re-infestation occurred in all the turtles 24–48 h after freshwater treatment. Formalin and chlorine treatments reduced the number of copepods (parasitic loads were estimated as being below 100 individuals/turtle), but infestation lingered in all the turtles. However after the second application of lufenuron, no copepods were detected on the turtles.

## Discussion

Ogawa et al. [16] described by the first time *B. manatorum* based on specimens collected from a captive loggerhead sea turtle, with discolored neck skin. The gut content of copepods displayed the similar gross appearance as scraped neck turtle skin, which led Ogawa [16] to suggest that *B. manatorum* could eat turtle skin. This suggestion was later supported by Badillo et al. [15], who found *B. manatorum* to be associated with skin lesions in one of the 52 turtles stranded on the western Mediterranean coast. Some *B. manatorum* specimens examined with scanning electron microscopy by these authors had pieces of tissue in their mouths, which closely resembled turtle skin. In contrast, Suárez-Morales et al. [20] found masses of *B. manatorum* arranged along natural skin folds on manatees, but with no associated lesions. This led these authors to suggest that, at least on these hosts, *B. manatorum* could behave as a commensal by feeding on suspended food particles. After scanning electronic microscopy, despite no genetic work has been conducted, Aznar et al. [18] pointed out the necessity of further attention on species complexity and possibly species variation along different described host.

The results from our study clearly indicated that *B. manatorum* individuals consume turtle skin. First, the lesions observed on the loggerhead sea turtle hatchlings were compatible with severe skin erosion. Second, the immunohistochemistry analysis showed that the pellet contained keratin. Experimental feeding trial results indicated that *B. manatorum* individuals readily consume skin flakes of sea turtles, but not algae, fish epidermis or baleen tissue [21]. Badillo et al. [15] wondered if *B. unisetus* could obtain energy from α-keratin of baleen plates of whales, from associated microscopic epibiota, or from both. Evidence from the present study confirmed that *B. manatorum* also selects microhabitats that are α-keratin-rich [25, 26]. Regardless of what the ultimate source of energy is, it seems clear that both species of *Balaenophilus* ingest host tissue and, with *B. manatorum*, this activity leads to mild to severe skin lesions in sea turtles.

Skin and shell lesions demonstrated the marked pathogenic action of *B. manatorum* in neonate loggerhead sea turtles kept in captivity. Open wounds can facilitate the action of secondary pathogens, particularly viruses, bacteria or fungi, and provoke homeostatic imbalance, which could complicate initial damage. In this outbreak, death of neonates was likely associated with water and electrolytic loss, stress and potential septicemia due to skin barrier alterations. One interesting question is to what extent are these effects related to captivity conditions? Obviously, neonates were exposed to high recruitment rates, but presumably also had an underdeveloped immune system. In fact, the late juvenile and adult turtles that were at the rescue center at the time of the outbreak were also exposed to infective stages of *B. manatorum*. However, these animals only carried moderate copepod loads and displayed no obvious signs of disease. The lesser skin thickness in neonates compared with the thicker skin of older animals could also exacerbate negative health effects of infestation by *B. manatorum*. Gross skin injuries have seldom been reported in juveniles and adults of *C. caretta*, and only mild signs of harm have been associated with heavy infestations [15, 16]. This raises the question as to whether no pathogenic effect of *B. manatorum* in manatees [20] could be related to a combination of the host’s very thick skin and low infection levels. Bite wound lesions as possible skin injuries cause [9] was discarded as turtles remained isolated each other; neither ulcerative dermatitis associated to immunosuppressed turtles [27] was rejected as lesions quickly improve after ectoparasite removal. Despite those findings, further control studies should be addressed to discern immunological sea turtle status effect on parasite-host relationship as well as predisposing environmental factors influencing copepod multiplication.

In addition to potential pathogenicity, the vector function of *B. manatorum* should be considered [11]. Chelonid herpesvirus 5 (ChHV5) has been identified as the likely etiological agent of fibropapillomatosis (FP) in sea turtles, an emerging infectious disease [28]. Some major aspects of FP epidemiology still remain unclear, but the implication of a vector in transmission has been hypothesized [29, 30]. Greenblatt et al. [30] found that the marine leeches of the genus *Ozobranchus* were candidates as FP vectors were based on the viral load determined by quantitative PCR. The possibility that *B. manatorum* could play a role as a potential secondary FP vector via skin lesions should be investigated.

How *B. manatorum* infested *C. caretta* neonates is an interesting question. Based on morphological evidence, Ogawa et al. [16] pointed out that this parasite’s whole life cycle can occur on the same host. This would suggest that close contact is necessary for transmission. However, recent swimming trials have indicated that copepodites IV and V, and *B. manatorum* adults, can perform fast directional swimming and could, therefore, switch hosts over short distances [21]. We suspect that some of the juvenile turtles in rehabilitation carried *B. manatorum* from the wild, and that the close proximity with neonates triggered host switching in the rearing facility. The *B. manatorum* prevalence among loggerhead sea turtles in the western Mediterranean Sea is over 80% [15], and all incoming turtles from the wild that were carried to the rescue center after the outbreak harbored *B. manatorum*.

The Lufenuron bath (added to the filtration system) proved an effective treatment against *B. manatorum*. This insect growth regulator, a phenylbenzoyl-urea that inhibits chitin synthesis, is used for the veterinary control of fleas on pets and cockroaches in houses as well as used for treating aquatic chitinous ectoparasites [24]. It is also used for pest control purposes in vegetables and fruits [31]. As lufenuron inhibits larval development, an additional agent is required to eliminate adults in the short term. In the present study, 10-min tap water baths had an immediate killing effect on *B. manatorum* adults. Chlorine and formalin were not effective for controlling infestation of *B. manatorum* at the doses used. Higher levels or more frequent application regimes might be effective, but possible adverse effects on turtles should also be taken into account.

## Conclusions

Our findings provide insight into potential pathogens in the wild to early life stages of sea turtles, where large gaps in our knowledge on natural threats appear. Admission quarantine protocols should be standard in aquaria, rehabilitation centers and conservational projects that manage head-starting or breeding programs. This study confirms the exceptional feeding behavior of *B. manatorum*, and opens up new lines of research to understand this interesting host-parasite system.

## Abbreviations

ABC: Avidin-biotin-peroxidase complex
*B. manatorum*: *Balaenophilus manatorum*
*B. unisetus*: *Balaenophilus unisetus*
ChHV5: Chelonid herpesvirus 5
FP: Fibropapillomatosis
H&E: Hematoxylin and eosin
PCR: Polymerase chain reaction
SD: Standard deviation
